# Synthesis of space-time wave packets using correlated frequency comb and spatial field

**DOI:** 10.1515/nanoph-2024-0771

**Published:** 2025-03-21

**Authors:** Alan E. Willner, Xinzhou Su, Yue Zuo, Yingning Wang, Zile Jiang, Amir Minoofar, Hongkun Lian, Zixun Zhao, Abdulrahman Alhaddad, Ruoyu Zeng

**Affiliations:** Department of Electrical and Computer Engineering and Department of Physics and Astronomy, 5116 University of Southern California, Los Angeles, CA 90089, USA; Department of Electrical and Computer Engineering, 5116 University of Southern California, Los Angeles, CA 90089, USA

**Keywords:** spatiotemporal, space-time wave packets, frequency comb, structured light

## Abstract

Shaping transverse degrees of freedom across different temporal frequency spectra has unlocked new possibilities for realizing a wide variety of novel spatiotemporal phenomena. In particular, using the discrete frequencies of optical frequency combs for spatiotemporal synthesis enables precise spatial separation and control of individual spectral lines, thereby facilitating the experimental generation of space-time wave packets (STWPs). This review explores the theoretical investigation and experimental demonstration of versatile STWPs synthesized using correlated frequency comb lines and spatial fields, including the following: (a) STWPs exhibiting dynamic evolution of spatial properties; (b) STWPs with customized group velocities; and (c) longitudinal control over the dynamic evolution of STWPs.

## 1 Introduction

Sculpting the complex electric field of light to achieve the precise manipulation of its properties is an exciting topic of photonics. For monochromatic light, this pursuit has given rise to the rapidly growing field of structured light, in which multiple spatial properties of light can be tailored, including amplitude, phase, polarization, and orbital angular momentum (OAM) [1], [2], [3]. It is possible to generate arbitrary optical fields in the transverse dimension by employing a set of orthogonal spatial modes, each characterized by a unique complex coefficient [2], [3]. This advanced structuring of light has paved the way for several potential applications, including optical communications [4], [5], high-resolution imaging [6], [7], and precision sensing [8], [9].

Expanding the monochromatic light field to broadband pulsed light unlocks even greater possibilities. Recent advancements in ultrafast pulse shaping have enabled spatiotemporal synthesis [10], [11], [12], [13], [14], [15], [16], [17], [18], [19], allowing for the simultaneous manipulation of both spatial and temporal degrees of freedom [20], [21], [22], [23], [24], [25], [26]. This approach has enabled the exploration of a wide range of unprecedented spatiotemporal phenomena [27], [28], [29], [30], [31], [32], [33], including the study of various families of space-time wave packets (STWPs). In these cases, distinct predetermined spatial field profiles are assigned to different temporal frequencies, resulting in correlated space-time light fields. This method has unveiled new dynamic behaviors in spatial properties [20], [27], [28], [29], [34], enabled the precise manipulation of group velocities and propagation in media [30], [35], [36], [37], [38], [39], [40], [41], generated spatiotemporal OAM evolution [42], [43], [44] and opened possibilities for potential applications [45], [46], [47], [48], [49], [50], [51], [52], [53], [54].

Specifically, spatiotemporal light fields can be decomposed into combinations of transverse spatial, longitudinal, and temporal frequency components [28], [29]. Temporal waveforms are intrinsically linked to their frequency components through Fourier transforms, and similar analogies apply to the spatial and longitudinal domains. In this regard, optical frequency combs, with their discrete, equally spaced frequency lines, emerge as suitable candidates for spatiotemporal synthesis. This enables precise spatial separation and control of individual spectral lines, facilitating the experimental generation and manipulation of STWPs [28], [55].

In this paper, we review recent advancements in the theoretical investigation and experimental demonstration of versatile STWPs synthesized using optical frequency combs and spatial modes. In Section 2, we discuss STWPs that exhibit the dynamic evolution of spatial properties. Section 3 focuses on STWPs with tailored group velocities, while Section 4 delves into longitudinal control over the dynamic evolution of STWPs. These developments highlight the exciting potential of structured light in both spatial and spatiotemporal dimensions. We note that because of space constraints, this review does not cover various other types of spatiotemporal phenomena, such as flying focus [37], [54], [56], [57], transverse OAM [58], [59], [60], [61], [62], [63], [64], [65], [66], [67], [68], [69], and toroidal pulses [70], [71], [72].

## 2 STWPs with dynamic evolution of spatial properties

### 2.1 Background and concept

One major difference between STWPs and spatially structured light pulses is that each frequency component carries a unique predesigned spatial field in the STWPs, while it carries the same field in conventional pulses. Consequently, the interference between these spatial fields in the STWPs experiences a dynamic evolution because of the temporal phase differences between these fields. Through careful design, STWPs can have a wide variety of temporal dynamic evolutions of their transverse spatial properties [28], [29], [73].

**Figure 1:**
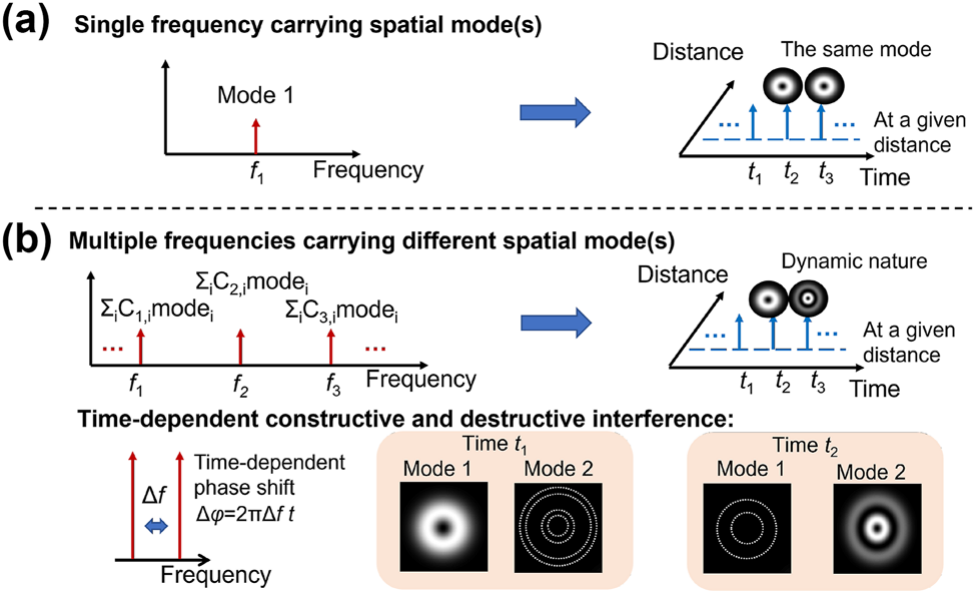
General concept of an STWP with dynamic evolution of its spatial profile. (a) The spatial profile of a single-frequency light field is static in time. (b) Correlating predetermined spatial fields on spectral frequencies leads to a time-varying property of the spatial evolution of the wave packet.

Figure 1 shows the basic concept and principle of STWPs with dynamically evolving spatial profiles. In the case of monochromatic beams, an arbitrary spatial distribution can be generated by a weighted combination of a certain set of orthogonal modes [74], as shown in Figure 1(a). One commonly used modal basis is Laguerre–Gaussian (LG) modes, which have two indices denoted by *ℓ* and *p*. However, such a monochromatic beam is static, as all the modes experience the same phase change with time. The temporal dynamic motion is made possible by introducing multiple frequencies, each assigned with designed spatial distributions (single mode or a weight combination of multiple modes), as shown in Figure 1(b). The frequency difference results in a time-dependent phase delay between the different mode combinations. This further induces constructive or destructive interference at different times, leading to the dynamic evolution of beam spatial distribution. Such dynamic STWPs can be experimentally synthesized using an optical frequency comb, which has multiple equally spaced frequency lines. Each frequency line can be assigned predetermined spatial profiles using spatial modulation techniques. At a fixed longitudinal distance, the transverse spatial profile of the STWP changes periodically with time, and the period corresponds to the spacing of the comb lines [28].

**Figure 2:**
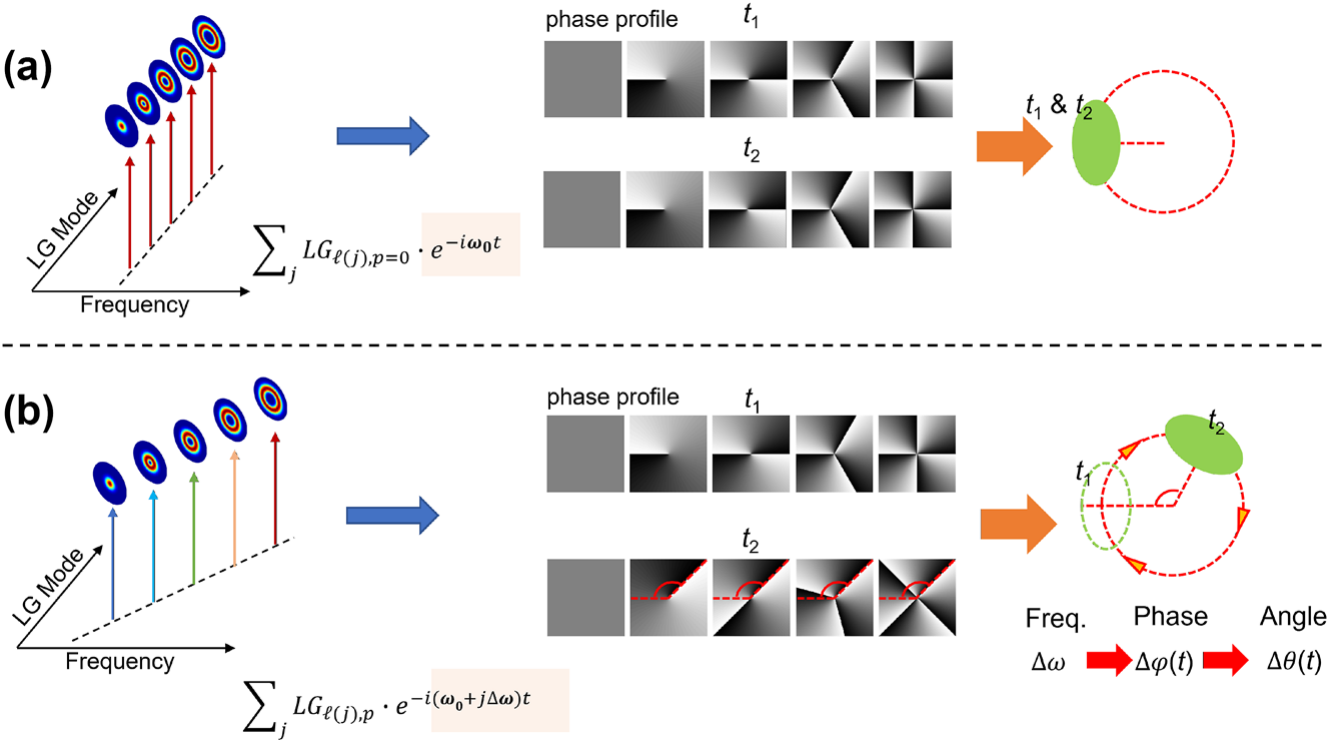
A specific example of an STWP with dynamic rotation of its spatial profile. (a) A monochromatic beam carrying multiple OAM modes shows a constructive interference in spatial profile at a certain azimuthal angle. This profile is static in time. (b) An STWP with a linear correlation between the OAM modes and the frequencies presents a dynamic rotation.

For instance, Figure 2 presents a basic example of a rotating STWP, which has been theoretically and experimentally investigated [47], [51], [73], [75], [76], [77], [78], [79]. When there are multiple LG modes on a single frequency, as shown in Figure 2(a), their phase profiles will interfere in the azimuthal spatial domain, resulting in a constructively interfered beam profile at a specific azimuthal angle. However, the intensity profile of the resulting beam is static because its phase front evolves at the same speed. Dynamic rotation is introduced when the OAM modes are correlated linearly with the frequencies, as shown in Figure 2(b). In this case, the time-dependent phase delay of each mode matches its own phase of the helical wavefront. As a result, the phase profiles of all modes have the same rotating speed, generating a beam with a rotating intensity profile. Except for this basic example, in this section, a few dynamic evolution properties of the spatial profiles are reviewed.

To experimentally generate and detect STWPs, a typical setup utilizes the integrated Kerr optical frequency comb with discrete equal-spaced comb lines [44], [55]. Several frequency lines with a spacing of 100 GHz are selected from the comb using a wavelength-selective switch (WSS). Each of them is subsequently modulated with a designed spatial profile using the spatial light modulator (SLM). The beams are then co-axially combined by another SLM to generate the STWP. For detection, the intensity and phase profiles of the STWPs are measured by a camera using spatiotemporal off-axis holography. Notably, this generation and detection technique can be adapted and utilized for various types of STWPs. Many of the subsequent experiments discussed in this paper share a similar experimental setup.

### 2.2 STWPs exhibiting two independent OAM

**Figure 3:**
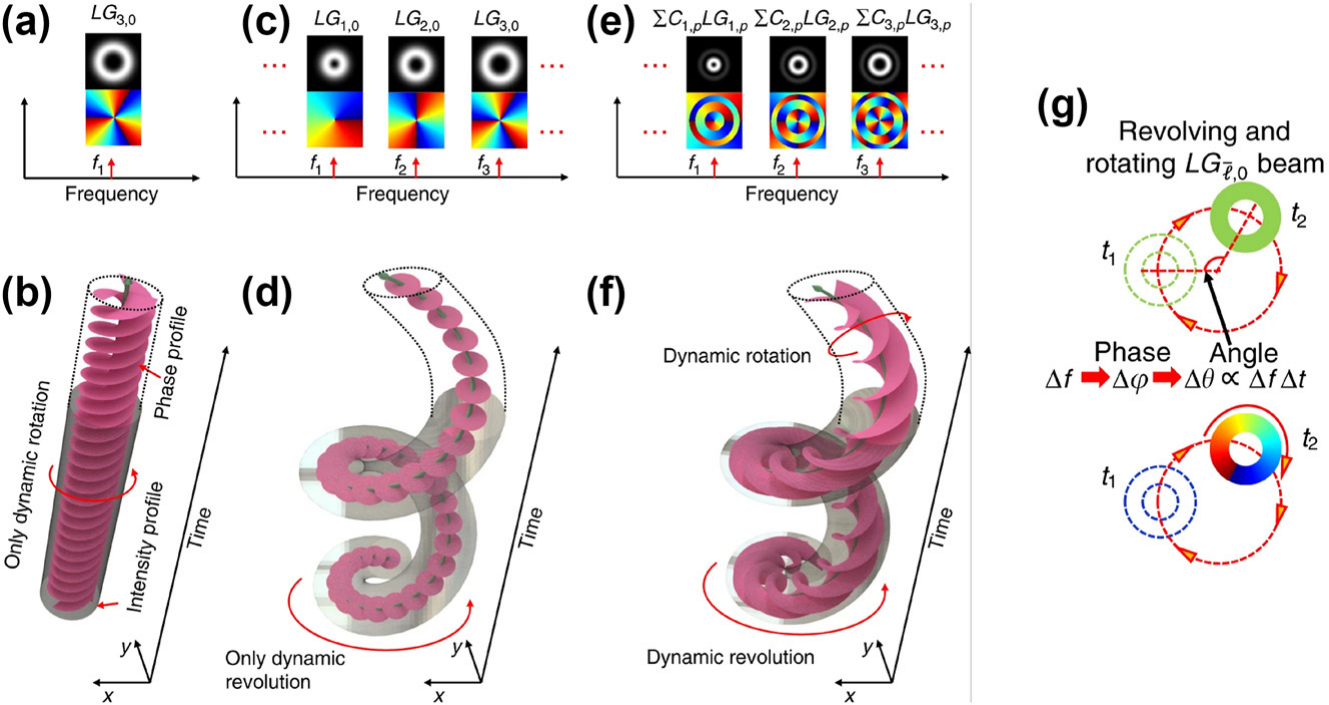
Concept of a spatiotemporal beam with both dynamic rotation and revolution. (a, b) An *LG*_3,0_ beam on a single frequency exhibits only rotation. (c, d) Multiple frequency lines, each assigned a single LG mode, exhibit only dynamic revolution. (e, f) Multiple frequency lines, each assigned multiple LG modes of different *ℓ* indices and *p* index, lead to both rotation and revolution. (g) Intensity and phase profiles of the rotating-revolving beam at different time instants [28]. Reprinted from Ref. [28], with permission from Springer Nature Publishing Group.

One early example of STWPs with dynamic spatial evolution proposed generating a spatiotemporal beam exhibiting two types of OAM (i.e., rotation and revolution with time) [28]. Figure 3 shows the concept of this rotating-revolving beam. As shown in Figure 3(a) and (b), a single LG mode on a single frequency will exhibit only a beam wavefront rotating around its own beam center. Figure 3(c) and (d) show the discussed dynamic rotation, which is the same as that shown in Figure 2. Here, we call this motion a dynamic revolution to distinguish it from the OAM wavefront rotation. Furthermore, when each frequency line is assigned multiple LG modes with a unique *ℓ* and multiple *p* indices, the resulting beam features both dynamics, as shown in Figure 3(e) and (f). Such beam dynamics are analogous to the motion of Earth, which has both rotation and revolution. The electric field at distance *z* = 0 is given by:

(1) $$\begin{array}{ll} & E\left(x,y,z=0,t\right)=LG_{\bar{\ell },\bar{p}}(x\cos\left(f_{\mathrm{rev}}t\right)-y\sin\left(f_{\mathrm{rev}}t\right)+R,\\ & \;\;x\sin\left(f_{\mathrm{rev}}t\right)+y\cos\left(f_{\mathrm{rev}}t\right),0;\omega_{0},w_{0})\exp\left(i\omega_{0}t\right) \end{array}$$

Here, *LG*_*ℓ*,*p*_(*x*, *y*, *z*; *ω*, *w*) denotes the electric field of the *LG*_*ℓ*,*p*_ mode with optical angular frequency *ω* and beam waist *w*. Eq. (1) describes an *LG*_*ℓ*,*p*_ beam dot exhibiting both rotation around its own beam center (characterized by the azimuthal index $\bar{\ell }$ ) and revolution around a central axis (characterized by the frequency *f*_*rev*_ and radius *R*), as illustrated in Figure 3(f). As mentioned above, such rotating-revolving LG beam can be constructed by combining multiple LG modes on different frequency lines, which is given by:

(2) $$E\left(x,y,0,t\right)=\overset{L}{\underset{\ell =-L}{\sum }}\overset{N}{\underset{p=0}{\sum }}C_{\ell ,p}LG_{\ell ,p}\left(x,y,0;\omega_{\ell },w_{0}\right)\exp\;\left(i\omega_{\ell }t\right)$$

Here, *C*_*ℓ*,*p*_ is the complex coefficient of mode *LG*_*ℓ*,*p*_. Therefore, $\sum_{p=0}^{N}C_{\ell ,p}LG_{\ell ,p}\left(x,y,0;\omega_{\ell },w_{0}\right)$ is a weighted combination of LG modes with multiple *p* indices assigned on the *ℓ*th frequency comb line, where *ω*_*ℓ*_ = *ω*_0_ + *ℓ*Δ*ω* denotes the optical angular frequency of that frequency line.

**Figure 4:**
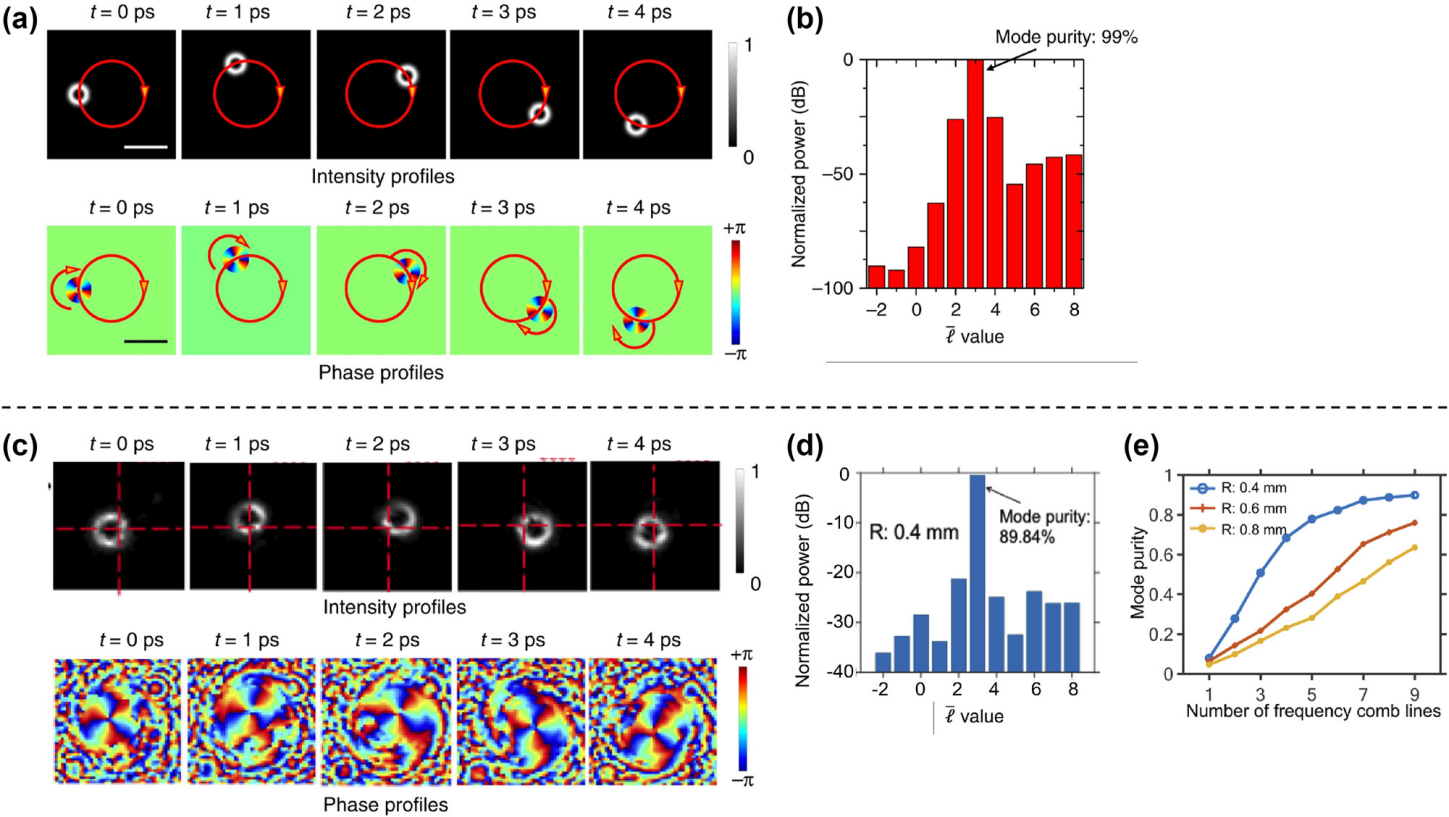
Simulation and experimental results of the rotating-revolving LG beam. (a) Simulated rotating-revolving *LG*_3,0_ beam with a revolution speed of 0.2 THz and radius of 0.75 mm. (b) Power distribution of the simulated beam on different $\bar{\ell }$ values. (c) Experimentally generated rotating-revolving *LG*_3,0_ beam with a revolution period of 5.2 ps and a radius of 0.4 mm. (d) Power distribution of the experimentally generated beam on different $\bar{\ell }$ values. (e) Relationship between modal purity and number of frequency lines under different revolution radii [28], [55]. (a, b) are reprinted from Ref. [28], with permission from Springer Nature Publishing Group. (c–e) are reprinted from Ref. [55], with permission from Optica Publishing Group.

As an example, a rotating-revolving *LG*_3,0_ beam is simulated in [28]. Figure 4(a) shows the evolution of the intensity and phase profiles of the beam with time at a fixed distance. The revolving radius is *R* = 0.75 mm, and the revolving speed is *f*_*rev*_ = 0.2 THz. The beam is constructed by 61 frequency lines (i.e., the *ℓ* index ranging from −30 to 30), while the *p* index ranges from 0 to 24. The mode purity of *LG*_3,0_ reaches 99 %, as shown in Figure 4(b).

Such a rotating-revolving LG beam was experimentally demonstrated in [55]. Figure 4(c)–(e) show the experimental results of a rotating-revolving *LG*_3,0_ beam with a revolution period of 5.2 ps and radius *R* = 0.4 mm. The beam dot evolves with time, exhibiting both rotation and revolution. The mode purity of the *LG*_3,0_ beam dot is measured to be 89.84 %.

The impact of frequency line number and revolving radius on beam quality is also studied, and the results are shown in Figure 4(e). The modal purity of the generated rotating-revolving beams decreases while using fewer frequency lines or increasing the revolving radius. This can potentially be explained using Fourier transform theory. A larger radius will cause the modes to be spatially distributed in a narrower azimuthal region, which requires more frequency components to achieve high modal purity.

### 2.3 Time-dependent beam radius

**Figure 5:**
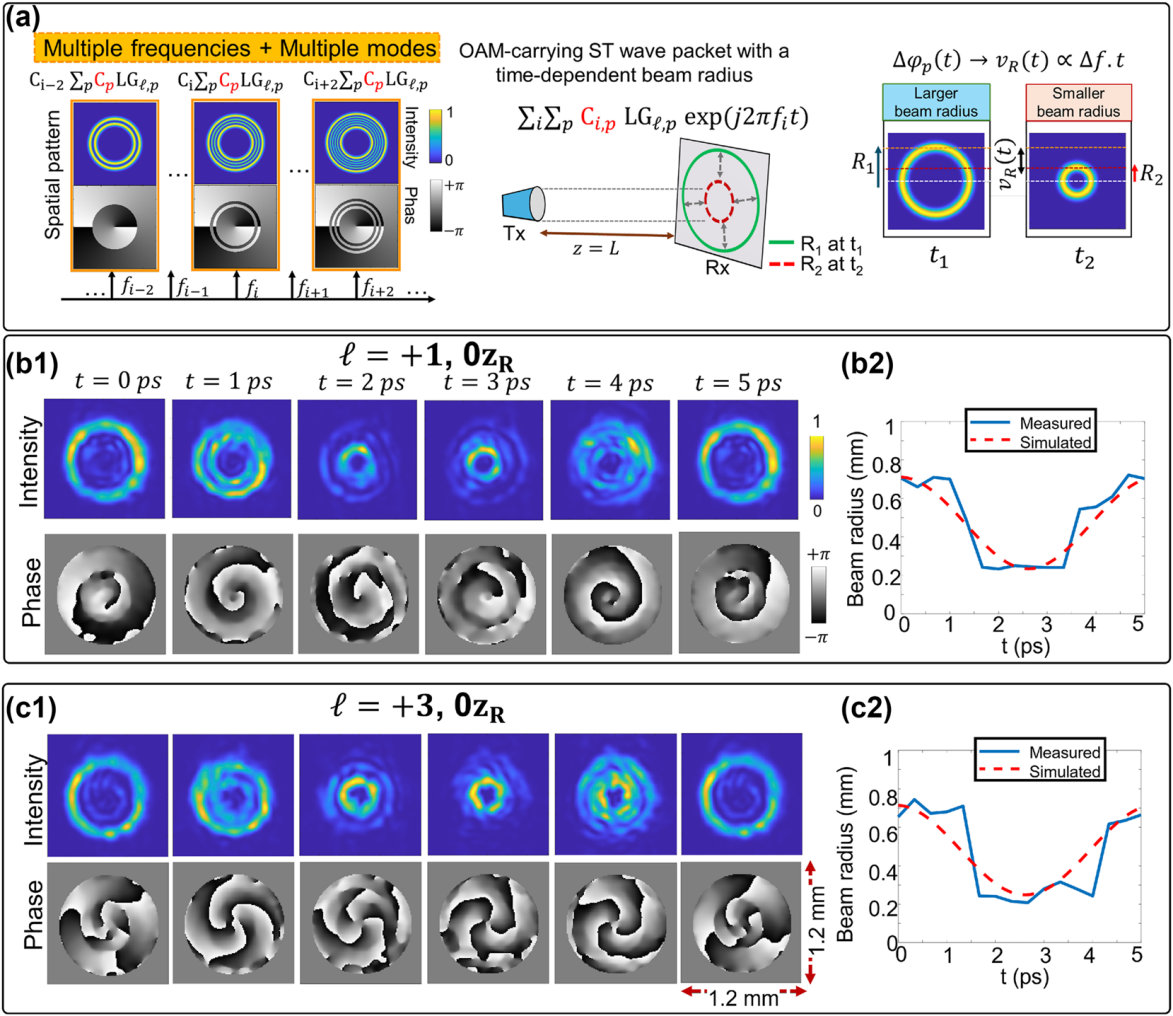
STWPs with time-dependent beam radius. (a) Multiple frequency lines carrying multiple spatial modes generate an OAM-carrying STWP with a time-dependent beam radius. (b, c) Intensity, phase profiles, and estimated beam radius of STWP with (b) *ℓ* = +1 and (c) *ℓ* = +3 [80]. Reprinted from Ref. [80], with permission from Optica Publishing Group.

STWPs can also be synthesized with a time-varying beam radius using a coherent combination of multiple spatial modes across frequencies [80]. Figure 5(a) illustrates the concept. To achieve a time-dependent beam radius, each frequency carries a designed spatial pattern with (i) a Hermite distribution along the radial axis [27], and (ii) a helical phase profile for the desired OAM order. These patterns are generated by combining LG modes with complex coefficients *C*_*p*_ determined through field overlap between the LG modes and the resultant spatial pattern. The frequencies *f*_*i*_ carrying different patterns are combined using Poisson formula coefficients *C*_*i*_ [27]. The electrical field can be expressed as follows:

(3) $$E\left(x,y,z,t\right)=\sum_{i}\sum_{p}C_{i,p}LG_{\ell ,p}\left(\rho ,\theta ,z;\omega_{i},w_{0}\right)\exp\;\left(j\omega_{i}t\right)$$

where *C*_*i*,*p*_ = *C*_*p*_ ⋅ *C*_*i*_ is a complex coefficient [80]. The time-dependent relative phase difference Δ*φ*_*p*_(*t*) between neighboring LG modes creates dynamic radial changes through interference. The intensity profiles oscillates at a rate *v*_*R*_(*t*), determined by frequency spacing Δ*f*.

This dynamic beam radius has also been experimentally demonstrated by synthesizing the spatial profiles of a frequency comb [80]. Figure 5(b) and (c) show the intensity and phase profiles and the estimated beam radius of experimentally generated STWPs for two cases: {*ℓ* = +1, *z* = 0} (b1, b2) and {*ℓ* = +3, *z* = 0} (c1, c2). The intensity profiles show radius oscillation with ∼5 ps (corresponding to ∼192 GHz frequency spacing), and the helical phase rotation follows the OAM order, as shown in Figure 5(b1) and (c1). Additionally, Figure 5(b2) and (c2) show the time dependence of the beam radius, with a beam waist of 0.3 mm. The radius oscillates between ∼0.24 mm and ∼0.68 mm.

### 2.4 Time-varying OAM

Time-varying OAM as a spatiotemporal phenomenon has attracted increased research interest in recent years. Time-varying OAM was first achieved through high harmonic conversion to ultraviolet (UV) frequencies [42]. Subsequent works have demonstrated the use of time-delayed sub-pulses with different OAM orders [21], [22]. Additionally, a different method has been developed, employing the frequency domain to shape the optical pulse [43].

**Figure 6:**
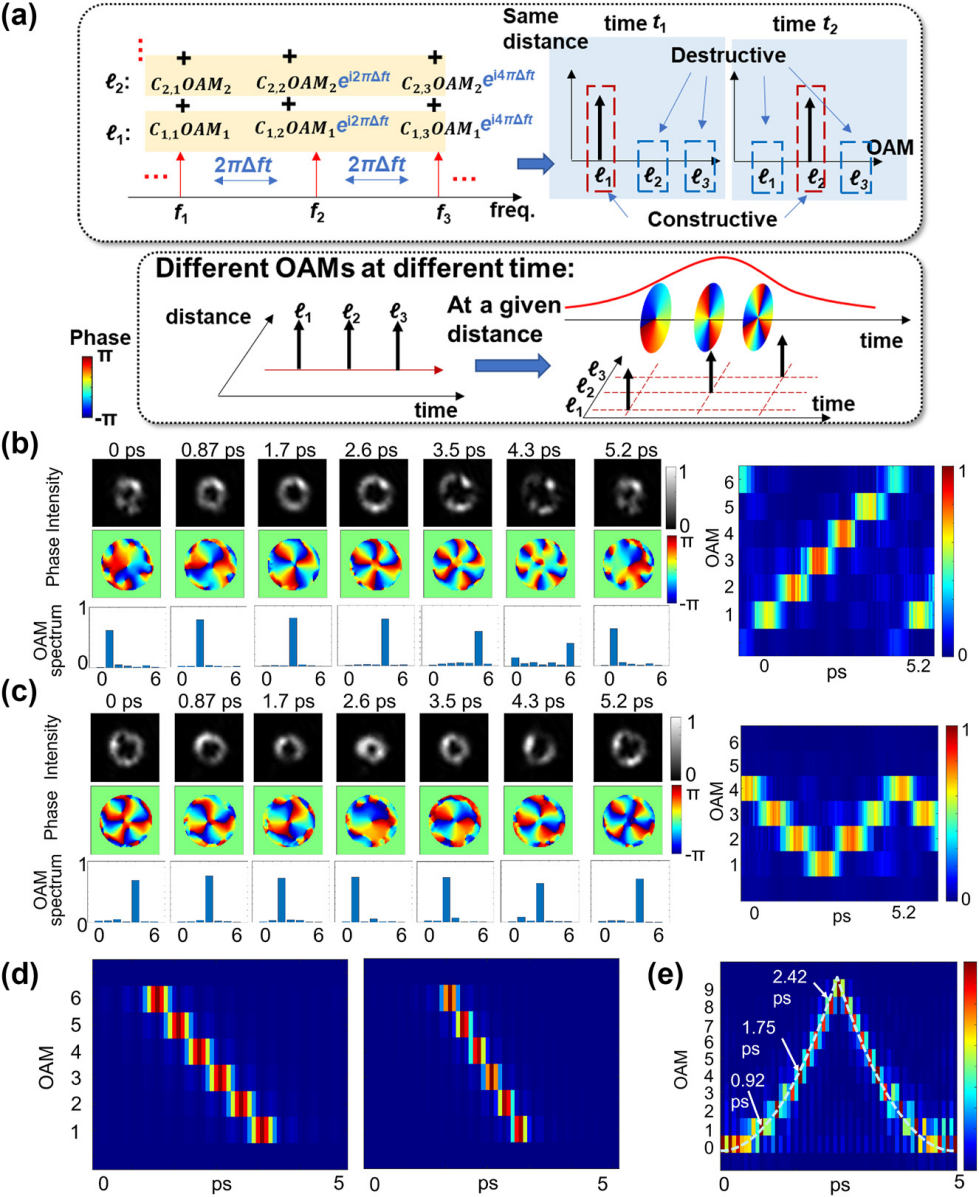
STWPs with time-varying OAM. (a) Concept of time-varying OAM. Different combinations of OAM orders are applied to different frequency lines, which results in an STWP with time-varying OAM. Experimental results of the STWPs with (b) OAM values increasing from +1 to +6, and (c) OAM values decreasing from +4 to +1 and increasing to +4 afterward. (d) Simulation results of the STWPs with 12 and 18 frequency lines. (e) Simulation results of the STWP carrying a roughly parabolically changing OAM value [43]. Reprinted from Ref. [43], with permission from Optica Publishing Group.

Figure 6(a) shows the concept of this method. Different combinations of OAM modes are applied to multiple distinct frequency lines. Such a combination will result in an STWP with varying OAM orders at different time instants at a given propagation distance. The frequency lines have a constant frequency separation, which results in constructive interference at some time instant and destructive interference at other time instants. Thus, the constructively combined OAMs will appear at different time instants, resulting in dynamically changing OAM orders of the STWP. The specific temporal distribution of OAM orders depends on the complex weights of the OAM modes applied to the distinct frequency lines. These weights can be calculated using the temporal Fourier transform of the desired distribution.

During the experiment, 6 frequency lines are used to generate the desired STWP. Figure 6(b) and (c) show the experimental results. Figure 6(b) demonstrates an STWP with six dynamically changing OAM orders from +1 to +6. The horizontal axis represents time, and the vertical axis represents the OAM order. A minimum mode purity of 70 % is reached, except for OAM + 6. Another interesting result is shown in Figure 6(c), in which the OAM orders decrease from +4 to +1 in the first half period and increase from +1 to +4 in the second half period. In addition to experiments, the effects of the number of frequency lines on the temporal pulse width are also investigated with simulations. Figure 6(d) shows the simulation results of the STWPs with different pulse widths, where the number of frequency lines is 12 and 18, respectively. As the number of frequency lines increases, the pulse width of the STWPs decreases. Figure 6(e) shows the simulated STWP carrying a nonlinearly varying OAM order that increases and decreases. Here, it is chosen to be a roughly parabolic function of time. These results prove the tunability of the STWPs and the proposed modulation method.

**Figure 7:**
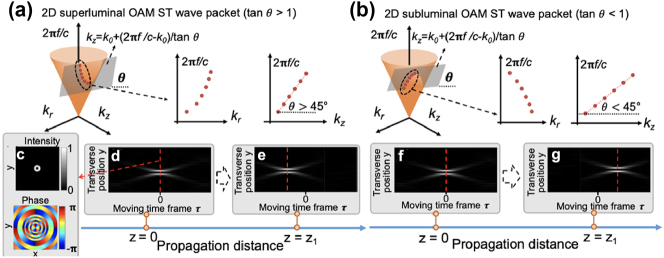
Concept for generating near-diffraction-free STWPs with controllable group velocity. Group velocity can be tuned by changing *θ*. As an example, (a) shows the space-time correlation of an OAM-carrying STWP with a superluminal group velocity. (c) The transverse profiles at the pulse intensity peak and (d, e) the intensity profiles of a superluminal STWP at two different *z* positions. (b) Shows the space-time correlation of an OAM-carrying STWP with a subluminal group velocity. (f, g) The intensity profiles of a subluminal STWP at two different *z* positions [81]. Reprinted from Ref. [81], with permission from Optica Publishing Group.

## 3 STWPs with controllable group velocity

### 3.1 Background and concept

Group velocity, defined as the propagation speed of a wave packet’s envelope, is one of the fundamental properties of an STWP [82], [83], [84], [85], [86], [87]. The group velocity of an STWP can also be manipulated by tailoring the spatial profile of each frequency line [30], [35], [36], [88], [89]. Figure 7(a) and (b) show the concept of OAM-carrying STWPs with tunable group velocity. The group velocity *v*_*g*_ of an STWP is given by:

(4) $$v_{g}=\frac{\partial \omega }{\partial k_{z}}$$

Here, *ω* and *k*_*z*_ denote the angular frequency and the longitudinal wavenumber of a beam, respectively. These two parameters, along with the radial wavenumber *k*_*r*_, follow the free-space dispersion relationship $k_{r}^{2}+k_{z}^{2}={\left(\frac{\omega }{c}\right)}^{2}$ , where *c* is the speed of light in free space. A Bessel beam is an OAM-carrying near-non-diffracting beam, of which both the OAM order and longitudinal wavenumber can be tuned by spatial modulation techniques (e.g., SLM). Therefore, a linear relationship between *ω* and *k*_*z*_ can be realized as:

(5) $$\omega_{i}=ck_{z_{0}}+c\left(k_{z_{i}},-,k_{z_{0}}\right)\tan\theta \;\left(i=-N,\ldots ,N\right)$$

Here, tan*θ* determines the proportional coefficient between *ω* and *k*_*z*_, which can be tuned by assigning specific *k*_*r*_ values on each frequency line. For example, an upward-opening quadratic relationship between *ω* and *k*_*r*_ will give *θ* > 45° (Figure 7(a)), while a downward-opening quadratic relationship will result in *θ* > 45° (Figure 7(b)). Based on Eqs. (4) and (5), the group velocity of the STWP is given by:

(6) $$v_{g}=c\tan\theta$$

Clearly, an STWP with *θ* > 45° and *θ* < 45° has superluminal and subluminal group velocity, respectively. Figure 7(d) shows the simulated transverse intensity and phase profiles of an OAM-carrying STWP with superluminal group velocity, which features a donut-like intensity distribution and helical wavefront. Figure 7(e) and (f) illustrate the *y* − *t* intensity profiles of this superluminal STWP at two different longitudinal locations. The wave packet shows an advance in time as compared to a luminal wave (corresponding to *τ* = 0 on the moving time frame) when propagating from *z* = 0 to *z* = *z*_1_. In contrast, Figure 7(g) and (h) illustrate the *y* − *t* intensity profiles of a subluminal STWP at the two same locations, where the opposite behavior (i.e., a time delay compared to luminal wave) is shown.

### 3.2 Synthesis of free-space STWPs with controllable group velocity using Bessel beams

Leveraging space-time correlations, several studies have demonstrated the generation of one-dimensional (1-D) diffraction- and dispersion-free STWPs with tunable group velocities in free space using plane waves [35], [88]. Generally, these wave packets are generated using SLMs with one dimension of the SLM modulating the temporal spectrum and the other dimension for one of the spatial spectra (e.g., the *x*-axis modulated while the *y*-axis freely propagates) [30]. Recently, with more advanced spatiotemporal synthesis, research has correlated 2-D Bessel–Gaussian beams and frequencies to generate 2-D STWPs with 2-D transverse spatial profiles carrying OAM and controllable group velocities [36], [81].

**Figure 8:**
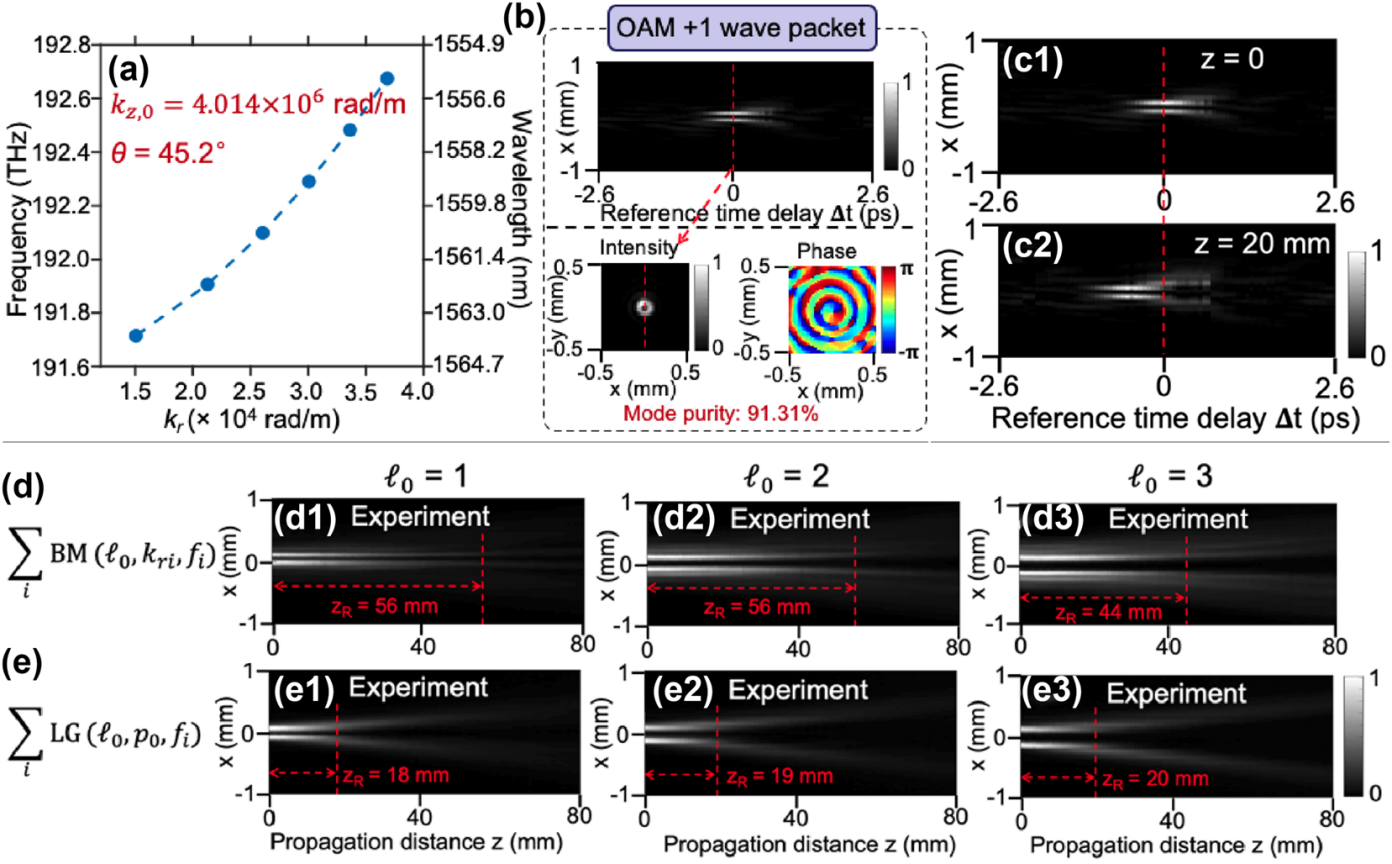
A 2-D STWP carrying OAM with tunable group velocity. (a) The designed space-time correlation with *θ* = 45.2°. (b) The intensity beam profile for the STWP carrying OAM + 1 with reference time delay and corresponding transverse profiles. The intensity profiles at (c1) *z* = 0 and (c2) *z* = 20 mm. (d) and (e) Show the time-averaged intensity profiles for STWP (OAM + 1, OAM + 2, and OAM + 3) and regular LG pulses at different *z* positions [81]. Reprinted from Ref. [81], with permission from Optica Publishing Group.

Figure 8(a) shows an example of the designed spatiotemporal spectrum for 2-D STWPs carrying OAM with *θ* = 45.2° [81]. The corresponding measurement results of the intensity profiles for OAM with *ℓ* = 1 at two positions are shown in Figure 8(c1) and (c2), respectively. A superluminal group velocity (*v*_*g*_ = 1.0069c) was observed, as evidenced by a temporal shift in the intensity peak.

Figure 8(d) shows the results of the time-averaged intensity profiles of the near-diffraction-free STWP (*ℓ* = 1, 2, 3) at different *z*-positions when *θ* = 45.2°. For comparison, LG-based OAM pulses were also studied, as shown in Figure 8(e). These LG-based pulses exhibit significantly greater diffraction over similar distances, confirming the superior near-diffraction-free performance of the Bessel-based STWPs.

**Figure 9:**
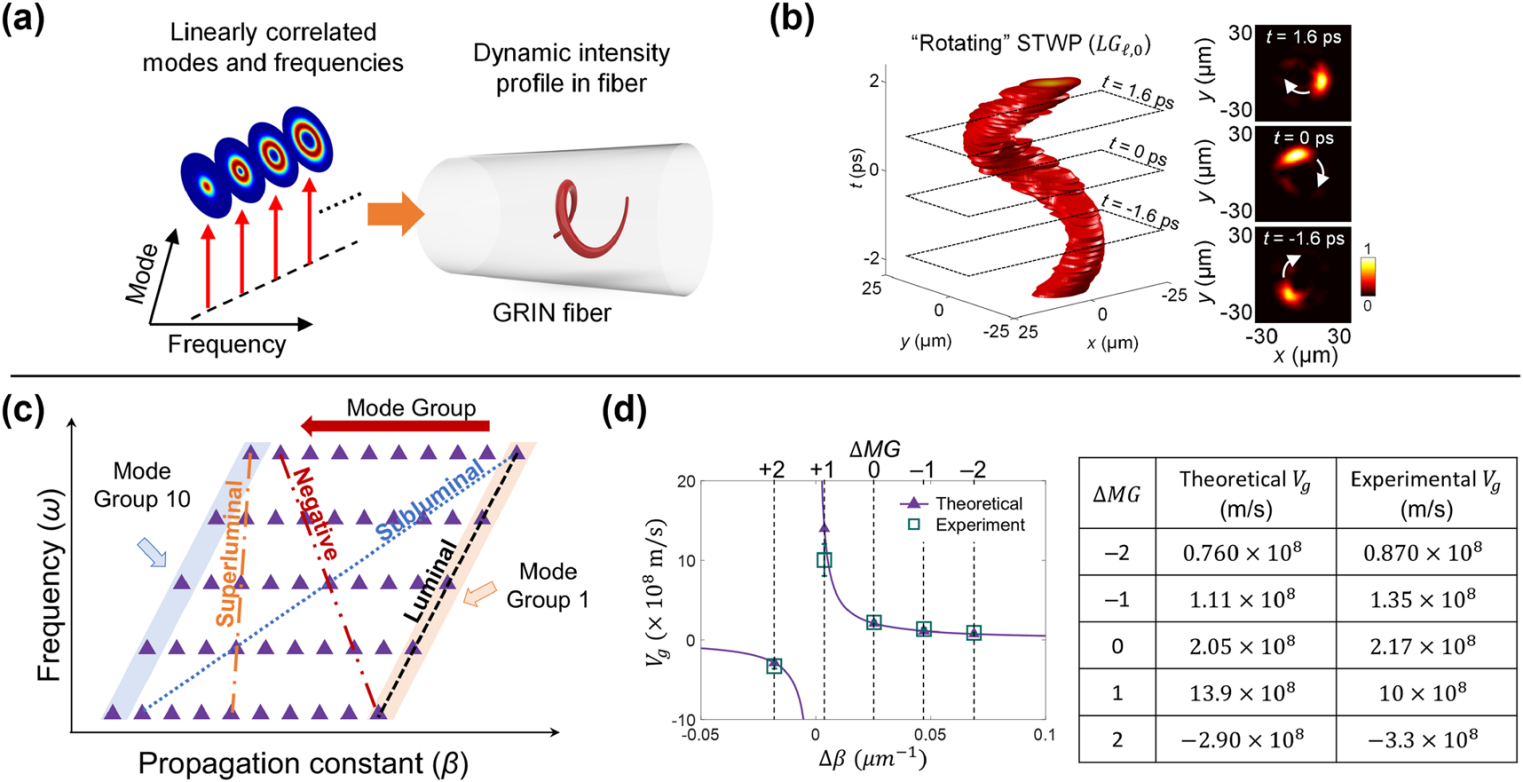
STWPs generated and propagated in MMFs. (a) Concept of dynamic motion: correlated spatial modes and optical frequencies of STWPs result in a temporally evolving transverse spatial profile in fiber. (b) Experimental transverse intensity profiles as a function of time. (c) Concept of group velocity control: in a comb-based STWP in GRIN fiber, *β* values are linearly correlated with frequencies. The slope of the *ω* − *β* curve can represent the *v*_*g*_. The *v*_*g*_ can be tailored to have superluminal, subluminal, or negative values. (b) Parabolic parameter of the fiber. (d) Theoretical and experimental *v*_*g*_ of STWPs with Δ*MG* of −2, −1, 0, 1, and 2 [90]. Reprinted from Ref. [90], with permission from Springer Nature Publishing Group.

### 3.3 STWPs synthesized in fibers

Common among all these examples of STWPs is their realization in a freely propagating field. Although initial demonstrations show guided STWPs in planar slab waveguides [91], [92], 1-D multimode slab waveguides [93], [94], and surface plasmon polaritons [95], [96], realizing STWPs with spatiotemporal properties in a fully confined optical waveguide or fiber remains a challenge [97], [98].

It has recently been reported that STWPs can be generated and propagated in a graded-index (GRIN) multimode fiber (MMF) with controllable dynamic motions and group velocities [90]. Unlike free-space systems in which an infinite set of spatial modes is available, propagation in fiber is only possible in terms of a finite number of guided modes whose propagation constants (*β*) are directly determined by the refractive index profile of the fiber itself [99]. Particularly, a GRIN MMF is a suitable candidate for fiber-based STWPs because of its unique dispersion relation. Guided modes in a GRIN MMF can be represented in different modal bases, such as LG modes. Fiber modes within the same modal group (MG) share a similar *β* value. Thus, the dynamic evolution of spatial profiles can be achieved with similar methods mentioned in Section 2.1. For group velocity control, a linear correlation between frequency, propagation constant *β*, and mode order for GRIN MMFs is utilized. The *v*_*g*_ of an STWP can be tailored by the dispersion relation d*ω*/d*β*. Leveraging the linear spacing between *β* for different MGs, the *v*_*g*_ of a comb-synthesized STWP can be controlled by a proper choice of MGs [73], [100], [101], [102].

Both dynamic evolution of spatial profiles and controllable group velocity have been experimentally demonstrated in a GRIN fiber [90]. As depicted in Figure 9(b), an STWP with temporal rotation is constructed using *LG*_*ℓ*,0_ modes when *ℓ* is linearly correlated with the 5 frequencies. The spatial intensity profile of the STWP rotates clockwise, and the rotating cycle equals the pulse period of 4.8 ps (i.e., 1/208 GHz). Figure 9(d) shows the theoretical and estimated experimental *v*_*g*_ as a function of mode group difference Δ*MG*. Specifically, Δ*MG* = 0 represents the Gaussian pulse corresponding to the luminal in MMF used as a reference. The experimental results generally match the theoretical predicted values. The group velocity can be tuned from subluminal to superluminal and negative values (e.g., 0.870, 1.35, 10, and −3.3 × 10^8^ m/s, respectively).

## 4 Longitudinal control of STWPs

### 4.1 Background and concept

In previous studies [21], [22], [23], [47], [79], [103], the temporal evolution of STWPs typically shares the same dynamic behavior at different longitudinal propagation distances. This is because the spatial field on each temporal frequency tends to have the same longitudinal propagation behavior. On the other hand, the propagation-dependent spatial variation of monochromatic beams has been demonstrated by tailoring multiple *k*_*z*_ values. As only a single temporal wavenumber *k*_*ω*_ is used, these monochromatic beams remain invariant in time [104], [105], [106], [107], [108], [109], [110], [111]. A novel 2-D control method was introduced in [44], allowing for the simultaneous manipulation of time-varying characteristics by tailored *k*_*ω*_ and longitudinal-varying properties by tailored *k*_*z*_. By combining both temporal and longitudinal degrees of freedom, these new STWP beams offer enhanced flexibility for spatiotemporal phenomenon generation.

**Figure 10:**
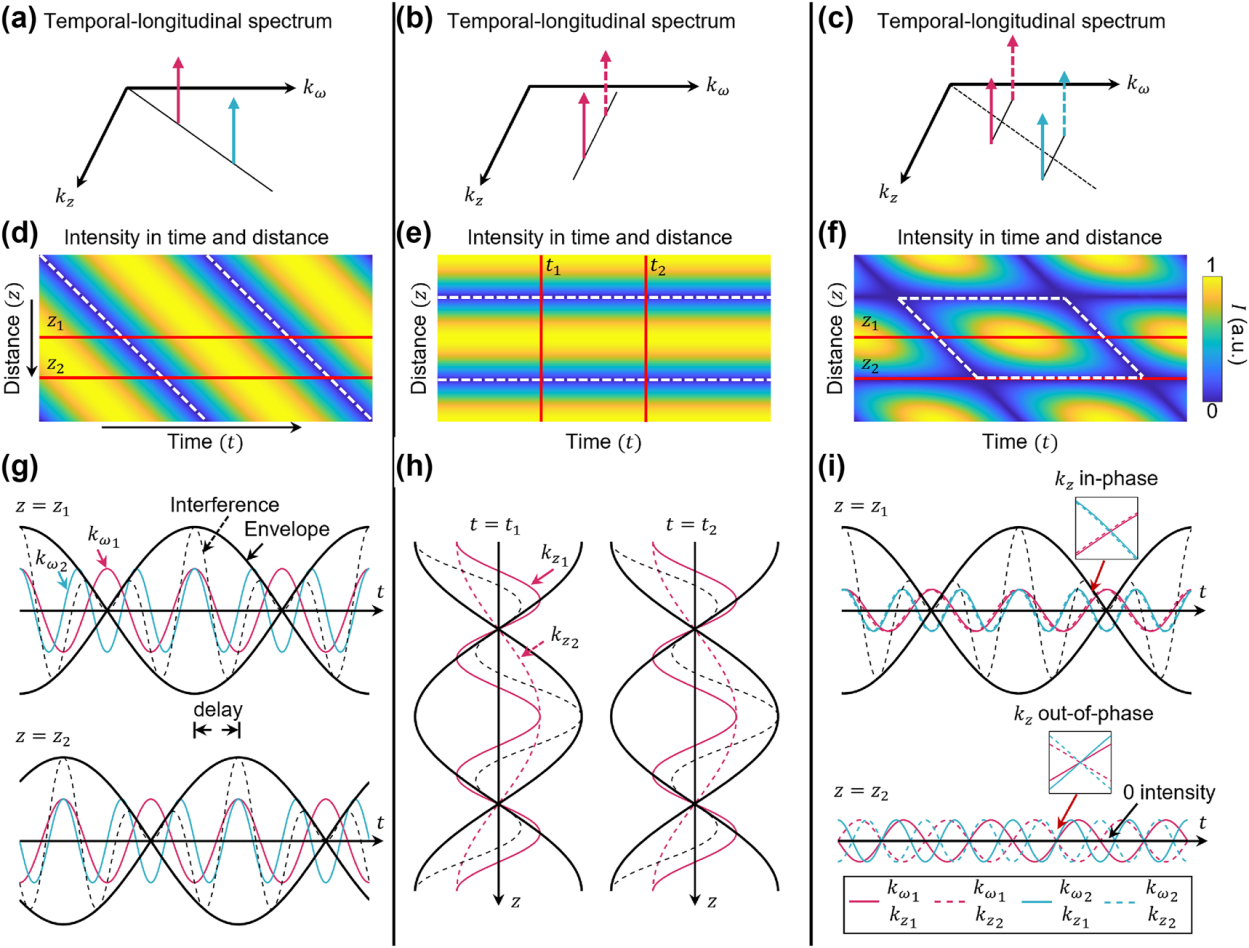
Interference patterns of coherent harmonic waves. (a) Superposition of two temporal frequencies, each with a longitudinal wavenumber. (b) Superposition of two longitudinal wavenumbers at the same temporal frequency. (c) Superposition of two temporal frequencies, each with two different longitudinal wavenumbers. (d–f) Intensity pattern of the superposed field as a function of time and propagation distance, with interference periods marked by white dashed lines. (g–i) Waveform interference at the red crosssections in (d–f). Red and blue lines show the real components of harmonic waves. Black dashed lines show their coherent sum, and black solid lines show the amplitude envelope [44]. Reprinted from Ref. [44], with permission from Optica Publishing Group.

Figure 10 illustrates the interference patterns using temporal or longitudinal spectral components, indicating the basic mechanism of temporal and longitudinal control. In Figure 10(a), the interference between two temporal frequencies creates a periodic temporal amplitude envelope, with a period $T=\frac{2\pi }{\Delta \omega }$ , where Δ*ω* is the angular frequency difference. In Figure 10(b), the interference between two longitudinal wavenumbers at a fixed temporal frequency produces a time-invariant longitudinal amplitude envelope with a period $L=\frac{2\pi }{\Delta k_{z}}$ , where Δ*k*_*z*_ is the wavenumber difference. Figure 10(c) shows the case in which both degrees of freedom are combined (i.e., two temporal frequencies associated with distinct longitudinal wavenumbers). The resulting 2-D interference pattern forms parallelogram-shaped structures in the time-distance plane.

To experimentally demonstrate this temporal and longitudinal control, Bessel–Gaussian beams $BG_{k_{\omega },k_{z},\ell }$ are utilized as the modal bases. The intensity and phase of a 0th-order wave packet can be controlled. The central waveform of the beam *s*(*z*, *t*), representing the complex amplitude and phase as a function of distance (*z*) and time (*t*), can be constructed by a superposition of spectral components as follows:

(7) $$s\left(z,t\right)=\sum_{n}\sum_{m}C_{n,m}BG_{k_{\omega_{n,m}},k_{z_{n,m}},\ell =0}\left(r=0,\theta =0;z,t\right)$$

where *r*, *θ*, and *z* represent the radius, angle, and distance in a cylindrical coordinate system. For the 2-D spectral design, a linear dispersion parameter (*α*) is introduced, coupling *k*_*ω*_ and *k*_*z*_. *k*_*ω*_ and *k*_*z*_ are chosen as discrete temporal and longitudinal frequency lines as:

(8) $$k_{\omega_{n,m}}=k_{\omega_{0}}+\frac{2\pi n}{T\cdot c},\;k_{z_{n,m}}=\frac{1}{\alpha }\cdot k_{\omega_{n,m}}+\frac{2\pi m}{L}$$

where *k*_*ω*,0_ and *k*_*z*,0_ are initial wavenumbers, and *T* and *L* are the temporal and longitudinal periods, respectively. The complex coefficient *C*_*n*,*m*_ corresponding to the *n*-th temporal and *m*-th longitudinal spectral components is derived through the Fourier relation as follows:

(9) $$C_{n,m}=\frac{1}{T\cdot L}\int_{0}^{T}\int_{0}^{L}s\left(z,t\right){\text{e}}^{\text{i}\left(\frac{2\pi }{T}nt-\frac{2\pi }{L}mz\right)}\;dt\;dz$$

This methodology extends naturally to various degrees of freedom, including (1) time- and longitudinal-varying polarization (TLV-Pol), in which a polarization-evolving beam is constructed as a combination of two orthogonal components *E*_*x*_ and *E*_*y*_, with independent amplitude and phase control [44]; (2) time- and longitudinal-varying OAM (TLV-OAM), in which beams are synthesized by superimposing sub-wave packets, each carrying distinct OAM values [44]; and (3) STWPs with longitudinally tailored dynamic rotation [112].

### 4.2 Temporal and longitudinal control of light spatial properties

**Figure 11:**
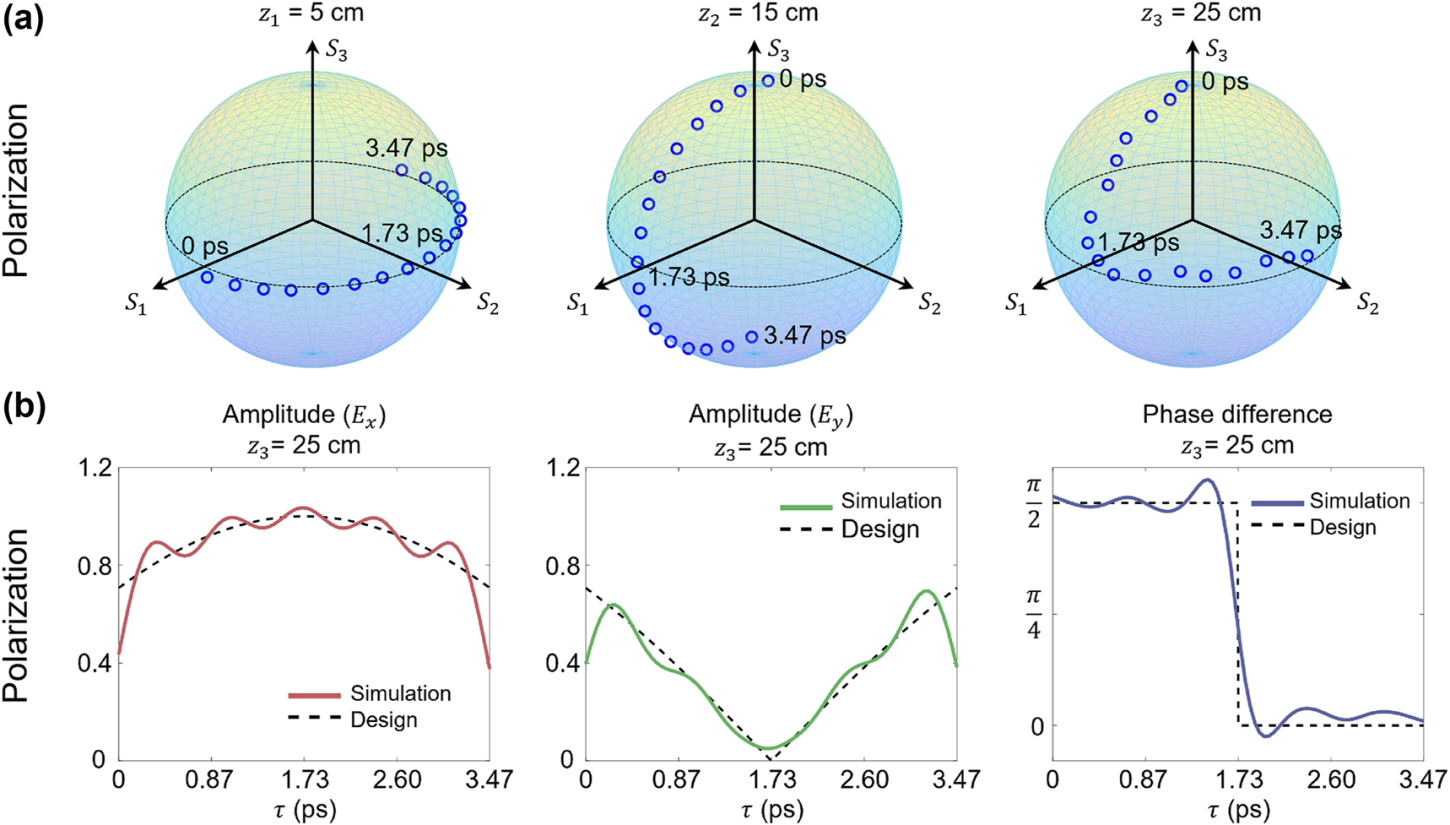
Simulation results of TLV-Pol, generated using the 2-D spatiotemporal synthesis method. (a) Polarization evolution in terms of time at three different locations, shown on the Poincaré sphere. (b) The ideal and simulated *E*_*x*_, *E*_*y*_ and their phase differences at distance *z*_3_ [44]. Reprinted from Ref. [44], with permission from Optica Publishing Group.

Temporal and longitudinal manipulation has been simulated and/or experimentally demonstrated [44], including on-axis intensity, polarization, and transverse spatial distribution (e.g., OAM), by using this 2-D spatiotemporal synthesis method. For instance, Figure 11 illustrates a simulated wave packet with dynamically evolving polarization along its propagation. By employing 15 (temporal) × 15 (longitudinal) spectral components, precise control over both the amplitude and phase difference between the $\hat{x}$ and $\hat{y}$ polarizations was achieved. Figure 11(a) shows the on-axis polarization evolution over time at three distinct locations. On a Poincaré sphere, the beam’s polarization state transitions along the equatorial plane for 0 ≤ *z* < 10 cm, shifts along the meridian plane for 10 ≤ *z* < 20 cm, and follows a combined trajectory of both for 20 ≤ *z* < 30 cm. The ideal and simulated amplitude waveforms of the two polarization components *E*_*x*_ and *E*_*y*_, along with their phase differences at *z*_3_ = 25 cm, are shown in Figure 11(b), demonstrating good agreement.

**Figure 12:**
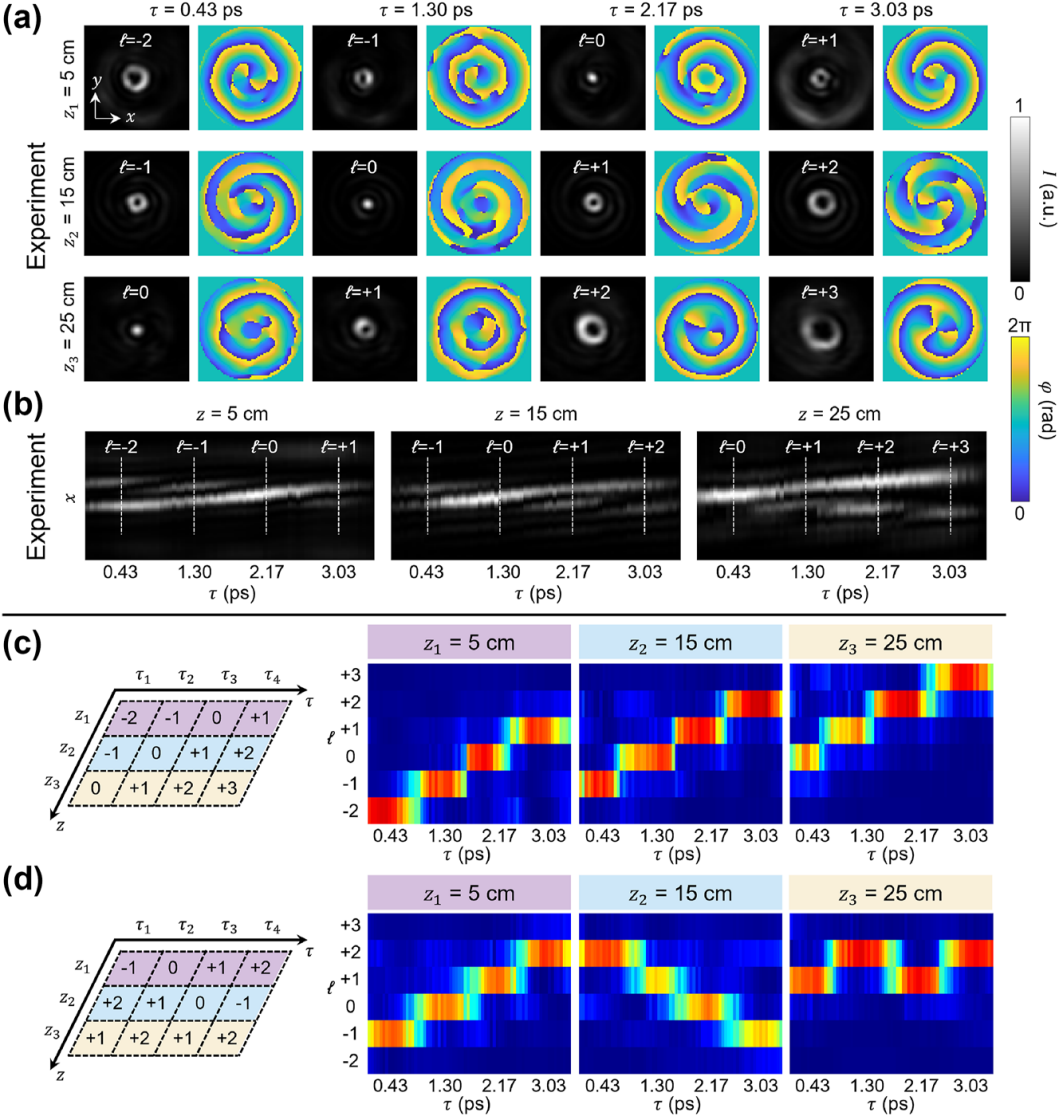
Experimental demonstration of a TLV-OAM beam. (a) Measured intensity and phase profiles of the wave packet. (b) Temporal evolution of the beam along the propagation direction. (c, d) Modal purity measurements at the spatial field center for different TLV-OAM beams, showcasing flexible tunability of the assigned OAM values [44]. Reprinted from Ref. [44], with permission from Optica Publishing Group.

Experimentally, a TLV-OAM beam was demonstrated by superposing multiple sub-STWPs that each carry a distinct topological charge value [44]. Using 6 (temporal) × 11 (longitudinal) spectral components, different OAM values were assigned at 4 time instants and 3 distances, with intervals of 0.87 ps in time and 10 cm in propagation distance. Figure 12(a) shows the experimentally measured intensity and phase profiles of a TLV-OAM wave packet, in which OAM values increase by +1 as time and distance advance. The designed OAM modes are shown in the beam’s center, while other OAM components spread power outside the central region because of destructive interference. Figure 12(b) shows the temporal evolution at three different positions. After isolation of the central lobe of the transverse field, the modal purity of the same beam was measured, as shown in Figure 12(c), achieving 75 %–90 % OAM purity.

This method can also be used to generate beams with complex OAM transitions by selecting specific modes for each temporal and longitudinal variation. Figure 12(d) shows an example of such flexible tunability, in which the OAM value exhibits increasing, decreasing and oscillating patterns at *z* = 5, 15, and 25 cm. Furthermore, this approach can be extended to generate multiplexed OAM states with variable weights, offering versatile control over spatiotemporal beam dynamics.

### 4.3 STWPs with longitudinally tailored dynamic rotation

Apart from controlling the basic transverse spatial properties, longitudinal control can be extended to other aforementioned dynamic evolutions, such as the dynamic rotation of spatial intensity profiles of STWPs. Recent advancements have experimentally demonstrated STWPs with tailored azimuthal motion of optical beams during propagation [112].

**Figure 13:**
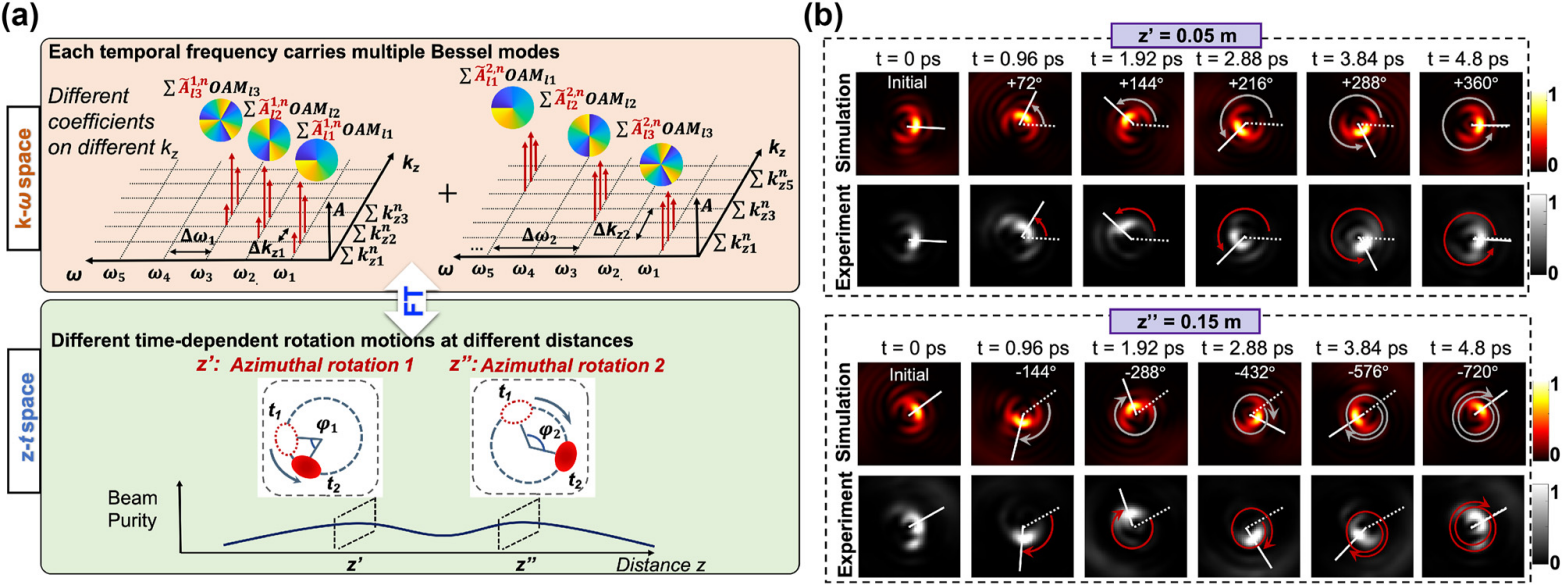
STWPs with longitudinally tailored dynamic rotation. (a) Concept for generating longitudinally transverse azimuthal rotations using a designed ST spectrum. (b) Simulated and experimentally measured beam transverse profiles at different time instants over two different distances (*z* = 0.05 and 0.15 m) [112]. Reprinted from Ref. [112], with permission from Optica Publishing Group.

Figure 13(a) illustrates the concept and principle of STWPs with range-dependent azimuthal rotational dynamics. This approach relies on coheretly combining multiple frequency lines, each modulated to carry superpositions of Bessel–Gaussian modes with distinct longitudinal wavenumbers. As previously discussed, the dynamic motion of the beam is governed by the frequency-OAM correlation. The rotation direction is controlled by the slope of the mode orders (positive or negative), while the rotation speed is adjusted by tuning the frequency line spacing. To achieve propagation-dependent rotation, multiple sub-wave packets are generated using the longitudinal control method, each having distinct dynamic rotation behaviors and localized at specific distance ranges.

Figure 13(b) presents the experimental and simulation results of longitudinally controllable azimuthal rotation dynamics. The beam transitions from a slow counterclockwise rotation with a period of 4.8 ps at *z* = 0.05 m to a faster clockwise rotation with a period of 2.4 ps at *z* = 0.15 m. The simulated and experimentally measured intensity profiles at different times and distances are in good agreement, validating the designed dynamic transitions.

## 5 Summary and discussion

This paper discusses and reviews recent advancements in the theoretical investigation and experimental demonstration of versatile STWPs synthesized using optical frequency combs, including (a) the dynamic evolution of spatial properties, (b) group velocity control, and (c) temporally and longitudinally tailored light properties.

Compared to experimental demonstration using a continuum spectrum, wave packets generated using frequency combs are generally limited to a few comb lines. A limited number of frequency lines can be accessed and processed in the current setup, where each frequency needs to be modulated on different locations of an SLM. This scheme can be potentially enhanced by exploiting alternative experimental approaches such as multi-plane light converters (MPLCs) [21] and metasurfaces [22]. Another potential approach might rely on dividing the entire spectrum into multiple sub-combs with fewer lines and recycling the spatial modulation for synthesis. The same frequency-mode assignment can be replicated for each sub-comb [83].

Although significant progress has been made in exploring spatiotemporal phenomena, further efforts are needed to translate these discoveries into practical applications. The unique properties of STWPs hold significant potential for a wide range of applications, including sensing, imaging, spectroscopy, and nonlinear interactions. With their customizable dynamically evolving spatial profiles and controllable group velocities, STWPs can enhance imaging resolution and offer additional dimensions of information, such as time-resolved spatial dynamics. In sensing applications, the tailored spatial and temporal properties can improve precision and sensitivity by enabling targeted interactions with the environment. Furthermore, the ability to manipulate group velocities and spatial profiles could play a crucial role in optimizing phase-matching conditions for nonlinear interactions, particularly in multimode waveguide systems. These advantages position STWPs as promising tools for advancing both fundamental research and practical technologies.
